# Secure Outsourcing of Matrix Determinant Computation under the Malicious Cloud

**DOI:** 10.3390/s21206821

**Published:** 2021-10-14

**Authors:** Mingyang Song, Yingpeng Sang

**Affiliations:** School of Computer Science and Engineering, Sun Yat-sen University, Guangzhou 510006, China; songmy5@mail2.sysu.edu.cn

**Keywords:** matrix determinant, secure outsourcing, cloud computing

## Abstract

Computing the determinant of large matrix is a time-consuming task, which is appearing more and more widely in science and engineering problems in the era of big data. Fortunately, cloud computing can provide large storage and computation resources, and thus, act as an ideal platform to complete computation outsourced from resource-constrained devices. However, cloud computing also causes security issues. For example, the curious cloud may spy on user privacy through outsourced data. The malicious cloud violating computing scripts, as well as cloud hardware failure, will lead to incorrect results. Therefore, we propose a secure outsourcing algorithm to compute the determinant of large matrix under the malicious cloud mode in this paper. The algorithm protects the privacy of the original matrix by applying row/column permutation and other transformations to the matrix. To resist malicious cheating on the computation tasks, a new verification method is utilized in our algorithm. Unlike previous algorithms that require multiple rounds of verification, our verification requires only one round without trading off the cheating detectability, which greatly reduces the local computation burden. Both theoretical and experimental analysis demonstrate that our algorithm achieves a better efficiency on local users than previous ones on various dimensions of matrices, without sacrificing the security requirements in terms of privacy protection and cheating detectability.

## 1. Introduction

The development of cloud computing provides great convenience to resource-constrained clients. They can outsource complex computations into the cloud by paying a fee. Computation outsourcing brings economic benefits to both resource-constrained clients and high-performance servers. Nevertheless, in practice, cloud servers are untrustworthy, which brings many security issues to computation outsourcing. According to [1], security has become a top issue that affects the business potential of cloud computing. The outsourced data usually contain private user information. The curious cloud may spy on user privacy through outsourced data. Besides, the malicious cloud may violate the computing scripts and return incorrect results to the client. Even without considering the maliciousness of the cloud, computing errors caused by the cloud hardware failure, software errors, etc. should also be detected by the client locally. In addition to these traditional security issues, with the development of smart phones, virtual assistants (VA) have been widely used in smart phones, which are vulnerable to malicious attacks; it uploads voice records to the cloud without the user’s knowledge or consent [2]. Therefore, cloud-based secure computation outsourcing algorithms have become hot topics of researches.

There are generally two types of security assumptions in cloud-based secure outsourcing algorithms [3]. The cloud that is assumed to be semihonest (or honest-but-curious) is only curious about the privacy contained in outsourced data. The cloud assumed to be malicious may firstly be semihonest, and then cause damage or forge results to sabotage the computation. Besides, in the above two security assumptions, the local computational burden of the client should be as low as possible. Otherwise, the efficiency benefit of outsourcing will be nullified. Therefore, the secure outsourcing algorithms under the malicious model require the considerations of efficiency, privacy protection, and cheating detectability.

In order to reduce the local computational burden using cloud computation outsourcing while protecting privacy and detecting false results, researchers have proposed secure outsourcing algorithms for many commonly used and complex computations, including the computation of matrix determinant. The computation of matrix determinant has appeared more and more widely in science and engineering problems recently. Many researches of medicine and biology use the matrix determinant for signal processing before using machine learning algorithms for practical classification tasks. For example, when machine learning algorithms are used for medical diagnosis, the data collected by the medical sensors are usually time series and contain private user information. Many researches divide the signal into several segments and fill them into matrices. Then, the determinants of matrices are considered as the feature values of the original signal. However, the computation of matrix determinant is still unaffordable for the sensors. They usually need to outsource matrix determinant computations to high-performance hardware.

To improve the detectability of cheating behaviors from the malicious cloud, all previous algorithms must increase the rounds of verification, which significantly increase the local computational burden of the client. Moreover, the currently known algorithm with the best privacy protection in [4] uses significantly more local computations than other algorithms. Therefore, we aim to propose a novel algorithm for secure outsourcing of matrix determinant computation, to solve the conflicts between the efficiency and security issues. The contributions of this paper are summarized as follows:

- We propose a secure outsourcing algorithm for the matrix determinant computation under the malicious cloud model, which can not only ensure the confidentiality of matrix, but also detect the forged results returned from the malicious cloud. We use the permutation, mix-row/mix-column, and split operations in our algorithm to protect privacy, which achieves the currently known lowest computation cost.
- We propose a one-round verification method in the proposed algorithm, which achieves a high cheating detectability. The malicious forged results can only escape our local verification with the probability of $\frac{1}{{\left(n!\right)}^{4}}$, given a matrix of n×n dimensions. In all the previous algorithms, the detectability of malicious forged results depends on the rounds of verification, and to achieve a high cheating detectability, multiple rounds of verification are required, which also brings high computational burden to the client. In the previous three algorithms [4,5,6], the succeeding probability of malicious forged results is $\frac{1}{2^{l}}$, where *l* is the number of verification rounds and recommended to be greater than 20.
- We conduct theoretical proofs of the correctness, efficiency, privacy protection, and cheating detectability for the proposed algorithm. Experimental results also demonstrate the superior efficiency of the proposed algorithm.

The rest of the paper is organized as follows: We introduce the related work and comparative analysis in Section 2. In Section 3, we introduce some background knowledge and the system model of our algorithm. Section 4 describes the proposed secure outsourcing algorithm for matrix determinant. We conduct the correctness, security, and complexity analysis of the proposed algorithm in Section 5. We evaluate the performance of our algorithm in Section 6. Finally, we conclude our work in Section 7.

## 2. Related Work and Comparative Analysis

Recently, the privacy and security issues in lightweight devices are widely concerned (e.g., the intrusion detection system on lightweight devices [7] and the secure computation outsourcing on resource-constrained devices). As we all know, although cloud computation outsourcing brings convenience to resource-constrained devices, it also causes privacy issues. Therefore, there are many researches on how to protect user privacy and verify the correctness of results when using cloud computing. From the perspective of the cloud, data access control mechanisms can be used to protect user privacy and ensure data availability. Kayes et al. [8] gave a survey on context-aware access control mechanisms (CAAC) during data management in cloud and fog networks. They also proposed a new generation of Fog-Based CAAC (FB-CAAC) framework for accessing data from distributed cloud data centers. When computations on the encrypted data are required, access control mechanisms are not enough. From the perspective of the client, Fully Homomorphic Encryption algorithms (FHE) [9,10,11,12] and Attribute-based Encryption algorithms (ABE) [13,14,15] have great application potential in cloud secure computation outsourcing, but their high computational complexities limit their practical applications, especially for resource-constrained devices. In addition, there are a large number of researches on the secure outsourcing algorithms for commonly-used and complex scientific computations (e.g., modular exponentiation [16,17], extended Euclidean [18], bilinear pairings [19,20,21], polynomial multiplication [22]).

There are many applications of matrix in the field of computer science, such as Digital Image Processing (DIP), computer graphics, computer geometry, Artificial Intelligence (AI), network communications, and so on. Thus, many computations of matrix are also commonly used and complex. However, some scientific computations of matrix have high computational complexities. Therefore, there are also many researches on secure outsourcing algorithms for computations of matrix. For example, the secure outsourcing algorithm for matrix multiplication has been widely studied [23,24,25,26]. In addition, Non-negative Matrix Factorization (NMF) is widely used in DIP, face recognition, text analysis, and other fields. Thus, there are many secure outsourcing algorithms for NMF [27,28,29,30]. Matrix inverse is also one of the most basic computations in large-scale data analysis. Computing the inverse of matrix on resource-constrained devices such as sensors is usually costly. Thus, there are also some secure outsourcing algorithms for matrix inverse [31,32,33]. Besides, in the field of machine learning, Singular Value Decomposition (SVD) has a wide range of applications. It can be used not only for feature decomposition in dimension reduction algorithms but also recommendation system, Natural Language Processing (NLP), and other fields. Securely outsourcing SVD to the cloud can greatly reduce the computation costs of the client. Chakan et al. proposed a secure outsourcing algorithm for SVD [34]. The local computational complexity of this algorithm is $O(n^{2})$ and the complexity of cloud is $O(n^{3})$. Chen et al. proposed a secure outsourcing scheme for SVD with less interactions between the client and the cloud [35].

Similar to the above computations of matrix, the determinant is also an important computation of matrix in the field of scientific and engineering. In the semihonest model, Kim et al. proposed a secure matrix determinant outsourcing method based on FHE [36]. Their scheme computes the determinant by the standard definition of matrix determinant, which results in a high computational burden of the client. Zong et al. introduced a division-free computational method for FHE-based secure matrix determinant computation outsourcing [37], which is significantly more efficient than the method in [36]. The above two algorithms only considered the privacy under the semihonest model.

However, in practice, the cloud may be malicious. To the best of our knowledge, the existing secure outsourcing algorithms for matrix determinant computation under the malicious model include [4,5,6]. In [5], Lei et al. used the block matrix and permutation techniques to protect privacy. In their algorithm, the client’s local computations include $\left(2+l\right)n^{2}+2m^{2}+4mn+2n+m$ multiplications, where *m* is the increase in dimension after encryption and *n* is the original dimension of matrix. Liu et al. proposed a new matrix determinant secure outsourcing algorithm using the permutation and mix-row/mix-column operations, which avoid the increase in matrix dimension during the encryption and reduces the number of local multiplications to $\left(2+l\right)n^{2}+3n$ [6]. Zhang et al. proposed a method that has better privacy [4]; however, because 8n times of elementary column/row transformations are involved in the process of encryption, it has a higher local computational burden than other algorithms, which require $\left(10+l\right)n^{2}+6n$ local multiplications. All the above three algorithms and our proposed algorithm have the same cloud computational complexity ($O(n^{2.373})$).

In our algorithm, in addition to the permutation and mix-row/mix-column operations, we used split operation to achieve a higher privacy. To prevent malicious cloud from forging computation results, the above three algorithms adopted the idea of Freivalds’ algorithm [38] with $ln^{2}$ computation costs that need to increase the frequency of verification (*l*) to improve the cheating detectability. *l* is greater than 20 at least in their works. In our proposed algorithm, only one round of verification is required, so there is no factor *l* in the computation cost. Table 1 demonstrates comparisons of our algorithm and the three existing algorithms from aspects of local multiplications, privacy protection level, and cheating detectability. Since multiplications dominate the local computation, local additions are omitted here and will be discussed later in Section 5. As we will discuss in Section 5, our proposed algorithm uses the lowest overall local computation cost to achieve a high detectability of result cheating (or a negligible probability of cheating success).

## 3. Preliminary

The notations and their implications used in this paper are shown in Table 2. This section will introduce some background knowledge of our algorithm.

### 3.1. System Model

The secure outsourcing algorithm for matrix determinant consists of the following five parts.

- KeyGen(λ) $\to (SK_{1},SK_{2})$: λ is a security parameter related to key generation. The generated key $SK_{1}$ is used to encrypt the input data, and $SK_{2}$ is used to verify and decrypt the returned results. Both $SK_{1}$ and $SK_{2}$ should be kept privately by the client *C*.
- Encrypt(*x*, $SK_{1}$) $\to (\sigma_{x})$: *x* is the input data. The client uses $SK_{1}$ to encrypt the input *x* and gets encrypted data $\sigma_{x}$. $\sigma_{x}$ is sent to the server *S* for computing.
- Compute(*f*, $\sigma_{x}$) $\to (\sigma_{y})$: *f* is a function given by the client. The server computes $\sigma_{y}$ using the given function *f* and encrypted data $\sigma_{x}$.
- Verify($\sigma_{y}$, $SK_{2}$) →(True/⊥): The client verifies the results returned from the cloud. If the $\sigma_{y}$ is valid, the output of this function is *Ture*. Otherwise, the output is ⊥.
- Decrypt($\sigma_{y}$, $SK_{2}$) →(y): The client uses the secret key $SK_{2}$ to decrypt $\sigma_{y}$ and obtains the result *y*.

**Figure 1:**
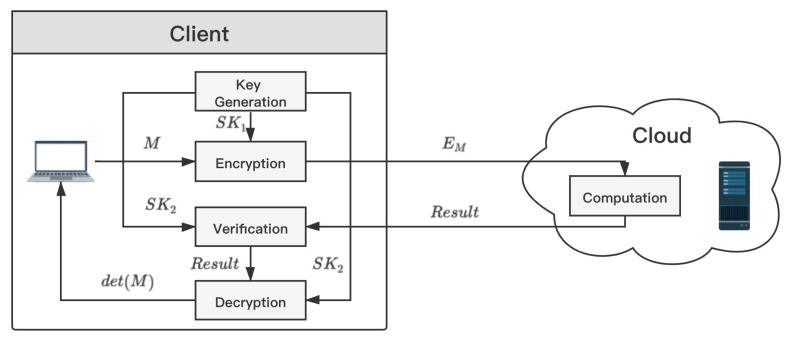
System model of secure outsourcing for matrix determinant computation.

The proposed algorithm is effective in the malicious cloud model. The malicious cloud can not only use its known information to infer the privacy of the client, but also maliciously forge false computation results to tamper with the whole algorithm. The system model of secure outsourcing for matrix determinant in this paper is shown as Figure 1.

### 3.2. Definitions of Correctness, Efficiency, and Security

When the key generation function *KeyGen*(λ) produces $\left(SK_{1},SK_{2}\right)$ satisfying $\forall x\in Domain_{define}$ ($Domain_{define}$ is the domain of input *x*), if *Encrypt*(*x*, $SK_{1}$) $\to (\sigma_{x})$ and *Compute*(*f*, $\sigma_{x}$) $\to (\sigma_{y})$, then y= *Decrypt*($\sigma_{y}$,$SK_{2}$). The outsourcing algorithm is *correct*.

A semihonest attacker may only be curious about the privacy contained in outsourced data. As shown in Equation (1), for any attacker *A* in the cloud server, if the probability of computing the secret input *x* with its known information ($\sigma_{x},f,\sigma_{y}$) is so small that it can be ignored in the polynomial time, the proposed secure outsourcing algorithm for matrix determinant is *privacy-protected*.

(1) $$Prob_{A}^{x}\left(\sigma_{x},f,\sigma_{y}\right)\to negli$$

A malicious attacker may cause damage or forge results to sabotage the computation. As shown in Equation (2), for any forged computation results (*forge*) returned from the cloud server, if the probability that the client (*C*) recognizes the forged results using the *Verify*($\sigma_{y}$,$SK_{2}$) function is infinitely close to 1, the proposed secure outsourcing algorithm for matrix determinant is *cheating-detected*, which means the *cheating detectability* of the algorithm is high, and the success probability of attacker’s result cheating is negligible.

(2) $$Prob_{C}^{forge}\left(Verify,SK_{2},\sigma_{y}\right)\to 1$$

If the local computation complexity of the client is substantially less than the computation complexity of the previous algorithm without outsourcing, the secure outsourcing algorithm for matrix determinant is *efficient*.

## 4. Secure Outsourcing of Matrix Determinant

In this section, we propose a secure outsourcing algorithm for matrix determinant. In comparison with the previous algorithms, our algorithm aims to improve the privacy protection ability and cheating detectability with less local computation costs. We use the permutation, mix-row/mix-column, and split operations in our algorithm to protect the privacy of matrix. Besides, we use a new result verification method to improve the cheating detectability, which is different from [4,5,6]. The main idea of the verification method is to ensure that at least the diagonal elements in the results of Low triangle and Up triangle (LU) decomposition returned from the cloud are correct. The security analysis in Section 5 will prove that it is more difficult for the forged results returned from the malicious cloud to pass this verification than the previous ones. The proposed algorithm is described as follows. The size of the input matrix is n×n in the rest of this section.

### 4.1. Key Generation

- The client needs to input a key space K. Eight diagonal matrices $\{P_{1},P_{2},P_{3},P_{4},Q_{1},$$Q_{2},Q_{3},Q_{4}\}$ are generated by selecting random nonzero values from K. Then, the client picks 8 random parameters $n_{1},n_{2},n_{3},n_{4},m_{1},m_{2},m_{3},m_{4}$ (lines 2 and 3 of Algorithm 1).
- The client computes $SK_{2}\leftarrow \left\{t_{1},t_{2},t_{3},t_{4}\right\}$ by lines 4–6 of Algorithm 1. It is easy to see that $t_{i}$ in $SK_{2}$ is the determinant of $P_{i}Q_{i}$.
- The client computes $SK_{1}$ by lines 7–14 of Algorithm 1.

**Algorithm 1** Procedure of Secret Key Generation
1:**function**KeyGen(λ)2:    Select random nonzero values from K to generate 8            diagonal matrices $\{P_{1},P_{2},P_{3},P_{4},Q_{1},Q_{2},$            $Q_{3},Q_{4}\}$3:    Pick 8 random parameters satisfying            $n\leq n_{1},n_{2},n_{3},n_{4},m_{1},m_{2},m_{3},m_{4}\leq 2n$4:    **for** i=1→4 **do**5:        $t_{i}\leftarrow {\left(-1\right)}^{n_{i}+m_{i}}\prod_{j=1}^{n}P_{i}\left(j,j\right)Q_{i}\left(j,j\right)$6:    **end for**7:    **for** i=1→4 **do**8:        **for** $j=1\to n_{i}$ **do**9:           Randomly select two rows of $P_{i}$ and exchange them.10:        **end for**11:        **for** $j=1\to m_{i}$ **do**12:           Randomly select two columns of $Q_{i}$ and exchange them.13:        **end for**14:    **end for**15:    **return** $SK_{1}\leftarrow \left\{P_{1},P_{2},P_{3},P_{4},Q_{1},Q_{2},Q_{3},Q_{4}\right\}$               $SK_{2}\leftarrow \left\{t_{1},t_{2},t_{3},t_{4}\right\}$16:
**end function**



In the KeyGen function, K can be ${\left\{0,1\right\}}^{\lambda }$, given a security parameter λ. From the analysis in Section 5, the number of elements in K is associated with the security of the algorithm. When λ=10, the probability that the cloud obtains the privacy input is less than $\frac{1}{2^{40}}$, which is negligible. Other ways of defining K are also applicable, as long as the number of elements in the set K is sufficient to resist the brute-force attack of the cloud.

(3) $$m_{i}^{\prime }+m_{i}^{\prime \prime }=m_{i}$$

(4) $$m_{j}^{\prime }+m_{j}^{\prime \prime }=m_{j}$$

### 4.2. Encryption

- While keeping the other rows, the client randomly splits the *i*th and *j*th rows of M to get four matrices $M_{1},M_{2},M_{3},M_{4}$. (lines 2–3 of Algorithm 2).
- The client computes $\sigma_{x}\leftarrow \left\{Y_{1},Y_{2},Y_{3},Y_{4}\right\}$ by lines 4–7 of Algorithm 2.

**Algorithm 2** Procedure of Encryption
1:**function**Encrypt($M={\left[m_{1},\ldots ,m_{n}\right]}^{T}$,$SK_{1}$)2:    Pick random i,j (1≤i,j≤n)3:    Construct matrices            $M_{1}={\left[m_{1},\ldots m_{i}^{\prime }\ldots ,m_{n}\right]}^{T}$;            $M_{2}={\left[m_{1},\ldots m_{i}^{\prime \prime }\ldots ,m_{n}\right]}^{T}$;            $M_{3}={\left[m_{1},\ldots m_{j}^{\prime }\ldots ,m_{n}\right]}^{T}$;            $M_{4}={\left[m_{1},\ldots m_{j}^{\prime \prime }\ldots ,m_{n}\right]}^{T}$;            satisfying Equations (3) and (4).4:    $Y_{1}\leftarrow P_{1}M_{1}Q_{1}$.5:    $Y_{2}\leftarrow P_{2}M_{2}Q_{2}$.6:    $Y_{3}\leftarrow P_{3}M_{3}Q_{3}$.7:    $Y_{4}\leftarrow P_{4}M_{4}Q_{4}$.8:    **return** $\sigma_{x}=\left\{Y_{1},Y_{2},Y_{3},Y_{4}\right\}$9:
**end function**



In the algorithm of *Encrypt*, the computations of matrices ($M_{1},M_{2},M_{3},M_{4}$) involve the generations of vectors $m_{i}^{\prime }$, $m_{i}^{\prime \prime }$, $m_{j}^{\prime }$, and $m_{j}^{\prime \prime }$. We can randomly pick *n* numbers as the elements of vector $m_{i}^{\prime }$ from K. Then, we can compute $m_{i}^{\prime \prime }=m_{i}-m_{i}^{\prime }$. In the same way, we can compute $m_{j}^{\prime }$ and $m_{j}^{\prime \prime }$. After computing the matrices $Y_{1},Y_{2},Y_{3},Y_{4}$, the client sends the matrices $Y_{1},Y_{2},Y_{3},Y_{4}$ to the cloud.

### 4.3. Computations of Server

- After receiving the matrices, the cloud computes the LU decomposition for $Y_{1}$, $Y_{2}$, $Y_{3}$, and $Y_{4}$ to get $L_{1},U_{1}$, $L_{2},U_{2},L_{3},U_{3}$, $L_{4},U_{4}$, where $f_{LU}$ is the LU decomposition function (lines 2–5 of Algorithm 3).
- The cloud computes determinants of $Y_{1},Y_{2},Y_{3},Y_{4}$ and obtains $r_{1},r_{2},r_{3},r_{4}$. (lines 6–9 of Algorithm 3).
- The cloud returns $\sigma_{y}=\left\{r_{1},r_{2},r_{3},r_{4},L_{1},U_{1},L_{3},U_{3}\right\}$ to the client.

(5) $$r_{1}\overset{?}{=}\prod_{i=1}^{n}L_{1}\left(i,i\right)U_{1}\left(i,i\right)$$

(6) $$r_{3}\overset{?}{=}\prod_{i=1}^{n}L_{3}\left(i,i\right)U_{3}\left(i,i\right)$$

(7) $$\frac{r_{1}}{t_{1}}+\frac{r_{2}}{t_{2}}\overset{?}{=}\frac{r_{3}}{t_{3}}+\frac{r_{4}}{t_{4}}$$

**Algorithm 3** Procedure of Computation
1:**function**Compute($\sigma_{x}$,$f_{LU}$)2:    $\left\{L_{1},U_{1}\right\}\leftarrow f_{LU}\left(Y_{1}\right)$.3:    $\left\{L_{2},U_{2}\right\}\leftarrow f_{LU}\left(Y_{2}\right)$.4:    $\left\{L_{3},U_{3}\right\}\leftarrow f_{LU}\left(Y_{3}\right)$.5:    $\left\{L_{4},U_{4}\right\}\leftarrow f_{LU}\left(Y_{4}\right)$.6:    $r_{1}\leftarrow \prod_{i=1}^{n}L_{1}\left(i,i\right)U_{1}\left(i,i\right)$.7:    $r_{2}\leftarrow \prod_{i=1}^{n}L_{2}\left(i,i\right)U_{2}\left(i,i\right)$.8:    $r_{3}\leftarrow \prod_{i=1}^{n}L_{3}\left(i,i\right)U_{3}\left(i,i\right)$.9:    $r_{4}\leftarrow \prod_{i=1}^{n}L_{4}\left(i,i\right)U_{4}\left(i,i\right)$.10:    **return** $\sigma_{y}=\left\{r_{1},r_{2},r_{3},r_{4},L_{1},L_{3},U_{1},U_{3}\right\}$11:
**end function**



### 4.4. Verification

- The client initializes the *flag* to *true*.
- The client performs the verifications of Equations (5)–(7). If any of them are invalid, the function returns ⊥ (lines 3–5 of Algorithm 4).
- The verifications in lines 6–17 verify all the diagonal elements in $L_{1},L_{3},U_{1},U_{3}$ at least once. If any verification is invalid, the function returns ⊥.
- If all the verifications are valid, the function returns *true*.

If the output of the *Verify* function is true, the clients executes the *Decrypt* function. Otherwise, the client rejects the results returned from the cloud.
**Algorithm 4** Procedure of Verification1:**function**Verify($\sigma_{y}$,$SK_{2}$)2:    flag←true3:    **if** any verification of Equations (5)–(7) is invalid **then**4:        flag←⊥; **return** flag.5:    **end if**6:    **for** i=1→n **do**7:        Pick j,k in [i,n] randomly.8:        **if** $Y_{1}\left(i,j\right)\neq l_{1}\left(i,-\right)\cdot u_{1}{\left(-,j\right)}^{T}$ or           $Y_{3}\left(i,k\right)\neq l_{3}\left(i,-\right)\cdot u_{3}{\left(-,k\right)}^{T}$ **then**9:           flag←⊥; **return** flag.10:        **end if**11:    **end for**12:    **for** j=1→n **do**13:        Pick i,k in [1,j] randomly.14:        **if** $Y_{1}\left(i,j\right)\neq l_{1}\left(i,-\right)\cdot {u_{1}\left(-,j\right)}^{T}$ or         $Y_{3}\left(k,j\right)\neq l_{3}\left(k,-\right)\cdot {u_{3}\left(-,j\right)}^{T}$ **then**15:           flag←⊥; **return** flag.16:        **end if**17:    **end for**18:    **return** flag19:**end function**

### 4.5. Decryption

- The client computes the result (Algorithm 5) $det\left(M\right)=y=\frac{r_{1}}{t_{1}}+\frac{r_{2}}{t_{2}}$.

**Algorithm 5** Procedure of Decryption
1:**function**Decrypt($\sigma_{y}$,$SK_{2}$)2:    $y\leftarrow \frac{r_{1}}{t_{1}}+\frac{r_{2}}{t_{2}}$3:    **return** *y*4:
**end function**



**Figure 2:**
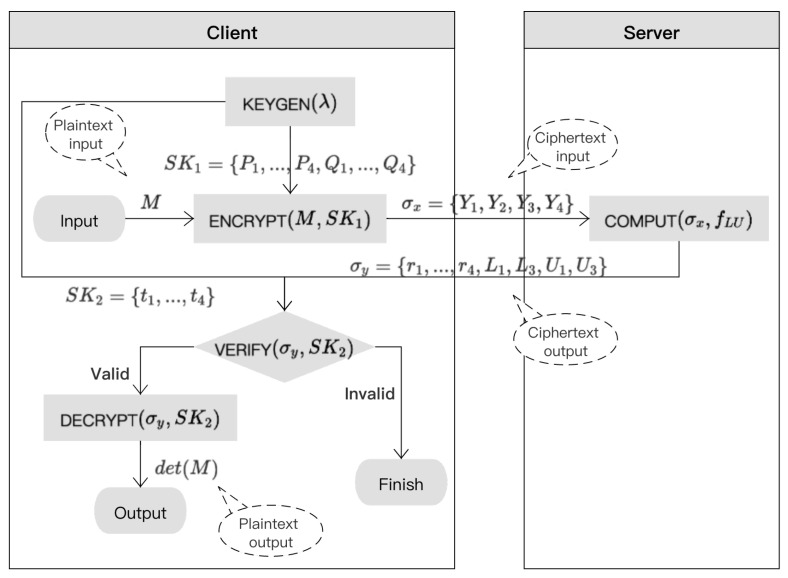
Flowchart of the secure outsourcing algorithm for matrix determinant computation.

Obviously, the input parameters of the *Verify* and *Decrypt* functions are the same. In fact, the result of the *Decrypt* function can be obtained during the execution of the *Verify* function in the proposed algorithm. We separate them into two parts for the sake of clarity and convenience to compare with the previous algorithms. The specific flowchart of the proposed algorithm is shown in Figure 2.

In the next section, the correctness, security, and computational complexity of the proposed algorithm will be analyzed.

## 5. Correctness, Security, and Computational Complexity Analysis

### 5.1. Correctness

**Theorem** **1.**  *The proposed secure outsourcing algorithm for matrix determinant is correct.*

**Proof of Theorem 1.**  When proving the correctness of a secure outsourcing algorithm, it is reasonable to believe that both the client and the cloud honestly follow the procedure of the algorithm. In the procedure of DECRYPT, the determinant of matrix is computed by

(8) $$det\left(M\right)=DECRYPT\left(\sigma_{y},SK_{2}\right)=\frac{r_{1}}{t_{1}}+\frac{r_{2}}{t_{2}}$$

where

(9) $$r1=det\left(Y_{1}\right)=det\left(P_{1}\right)det\left(M_{1}\right)det\left(Q_{1}\right)$$

(10) $$r2=det\left(Y_{2}\right)=det\left(P_{2}\right)det\left(M_{2}\right)det\left(Q_{2}\right)$$

According to the function KEYGEN, we can obtain

(11) $$t1=det\left(P_{1}\right)det\left(Q_{1}\right)$$

(12) $$t2=det\left(P_{2}\right)det\left(Q_{2}\right)$$

Thus, we can obtain

(13) $$\begin{array}{cc} det(M) & =det\left(M_{1}\right)+det\left(M_{2}\right)\\ \; & =\frac{det\left(P_{1}\right)det\left(M_{1}\right)det\left(Q_{1}\right)}{det\left(P_{1}\right)det\left(Q_{1}\right)}+\frac{det\left(P_{2}\right)det\left(M_{2}\right)det\left(Q_{2}\right)}{det\left(P_{2}\right)det\left(Q_{2}\right)}\\ \; & =\frac{det(Y_{1})}{det\left(P_{1}\right)det\left(Q_{1}\right)}+\frac{det(Y_{2})}{det\left(P_{2}\right)det\left(Q_{2}\right)}\\ \; & =\frac{r_{1}}{t_{1}}+\frac{r_{2}}{t_{2}} \end{array}$$

Finally, Equation (8) is proved. This implies that the function DECRYPT always yields the correct determinant and the proposed algorithm is correct. □

### 5.2. Computational Complexity

We analyze the computational complexities of the client and the cloud in this section. The KEYGEN function, ENCRYPT function, VERIFY function, and DECRYPT function are executed by the client. The COMPUTE function is executed by the cloud. We first analyze the computational complexity of the client.

**Theorem** **2.**  *The computational complexity of the client side of the proposed secure outsourcing algorithm for matrix determinant is $O(n^{2})$.*

**Proof of Theorem 2.**  The operations involved in the KEYGEN function include multiplication and mix-row/mix-column, where multiplications and additions need to be computed. The computations in lines 4–6 require a total of 8n multiplications. The computations in lines 7–14 and line 5 require a total of 8n+4 multiplications.

The ENCRYPT function involves matrix split operations and matrix multiplications. Obviously, the matrix split operation needs only 2n subtractions. The major computations are lines 4–7. Since $P_{1},P_{2},P_{3},P_{4},Q_{1},Q_{2},Q_{3}$, and $Q_{4}$ are obtained by randomly swapping rows/columns of diagonal matrices, the computations of $Y_{1},Y_{2},Y_{3}$, and $Y_{4}$ require a total of $8n^{2}$ multiplications; therefore, the ENCRYPT function requires a total of $8n^{2}$ multiplications and 2n subtractions.

In the VERIFY function, the verifications of Equations (5)–(7) require 4n multiplications. The verifications of lines 6–17 compute 4n times of the vector’s inner product, which require $4n^{2}$ additions and multiplications. The verification in Equation (7) requires 2 additions. Therefore, the VERIFY function requires $4n^{2}+4n$ multiplications and $4n^{2}+2$ additions.

Two divisions and one addition are required in DECRYPT. Thus, it is easy to see that the computational complexity of the DECRYPT function is O(1).

In conclusion, the client needs to undertake a total of $12n^{2}+12n$ multiplications and $4n^{2}+10n+7$ additions. Therefore, the computational complexity of the client side of the proposed algorithm is $O(n^{2})$. □

In Table 3, the number of multiplications required by every part of the proposed algorithm is demonstrated and compared with three existing algorithms from [4,5,6]. In Table 4, the numbers of additions required by the proposed algorithm are listed and compared in the same way as Table 3. Although the complexity of the *KEYGEN* and *ENCRYPT* is higher than the compared schemes [5,6], the complexities of *VERIFY* and *DECRYPT* in the compared schemes are higher than that of the proposed algorithm. According to [4,5,6], to improve security, the value of *l* is usually greater than 20 and the value of *m* is usually greater than 100, where *l* is the frequency of verification and *m* is the increase of the dimension after encryption. In fact, when *l* in the compared algorithms is set to 20, the security of those algorithms are very poor, which is equivalent to the security of the proposed algorithm running on a matrix of dimensions 4×4. When pursuing higher security, the local computational cost of the compared algorithms will be significantly higher than the algorithm in this paper. Therefore, it is easy to see that the proposed algorithm has the lowest local computational burden.

**Theorem** **3.**  *The computational complexity of the server side of the proposed secure outsourcing algorithm for matrix determinant is $O(n^{2.373})$.*

**Proof of Theorem 3.**  Only the COMPUTE function is performed by the server side. Four iterations of LU decomposition computations and 8n multiplications are required in Algorithm 3. If the server supposes the fastest LU decomposition algorithm (e.g., Williams’ algorithm [39]), the computational overhead for the cloud side can be reduced to $O(n^{2.373})$, which has been proven in [5]. □

In comparison with the previous algorithms, the specific theoretical performance of the proposed algorithm is shown in Table 5. The nonoutsourcing algorithm for matrix determinant is $O(n^{2.373})$ using the fast LU decomposition method in paper [39]. Obviously, the local computation complexity of the client ($O(n^{2})$) is substantially less than the computation complexity of nonoutsourcing ($O(n^{2.373})$).

### 5.3. Security

A cloud server can be a passive or active attacker. Next, we will analyze the security of the proposed algorithm against both passive and active attacks.

#### 5.3.1. Privacy against Passive Attacks

A passive attacker (semihonest) follows the procedure of the algorithm while exploiting the intermediate information to breach the privacy of matrix.

**Theorem** **4.**  *The proposed secure outsourcing algorithm for matrix determinant is privacy-protected.*

**Proof of Theorem 4.**  It is easy to see that the methods of encrypting matrices $M_{1},M_{2},M_{3}$, and $M_{4}$ are consistent. The privacy input matrix M can be obtained by computing $\left\{M_{1},M_{2}\right\}$ or $\left\{M_{3},M_{4}\right\}$. We take $\left\{M_{1},M_{2}\right\}$ as an example to prove that the proposed algorithm is privacy-protected. As $Y_{1}$ and $Y_{2}$ are visible to the attacker, to restore $\left\{M_{1},M_{2}\right\}$, the attacker needs to guess $P_{1}$, $Q_{1}$, $P_{2}$, and $Q_{2}$. Then, the attacker uses the inverse matrices of $P_{1}$, $Q_{1}$, $P_{2}$, and $Q_{2}$ to restore $\left\{M_{1},M_{2}\right\}$. When generating the original diagonal matrices $P_{1}$, $Q_{1}$, $P_{2}$, and $Q_{2}$ in the KEYGEN function, a total of 4n elements are selected from the key space K$={\left\{1,0\right\}}^{\lambda }$. The probability of attacker *A* correctly guessing the 4n elements is $\frac{1}{{\left(2^{\lambda }\right)}^{4n}}$. Besides, from the perspective of the attacker, as long as the frequencies of mix-rows/mix-columns ($n_{1},n_{2},m_{1},m_{2}$) are large enough, it is equivalent to repositioning the *n* nonzero elements of the diagonal matrix ($P_{1}$, $Q_{1}$, $P_{2}$, $Q_{2}$) to ensure that all rows/columns of the new matrix have only one element. Thus, through the mix-row/mix-column operations in lines 7–14 of Algorithm 1, the attacker successfully guessing any matrix requires n! attempts. Therefore, the passive attacker should make ${\left(n!\right)}^{4}{\left(2^{\lambda }\right)}^{4n}$ brute-force guesses to obtain $\left\{M_{1},M_{2}\right\}$. Then, the attacker can easily compute M. The probability that the attacker *A* obtains the secret input M is shown at Equation (14). When either the size of the matrix or the key space K is large enough, the value of $Prob_{A}^{M}$ will be so small that it can be ignored.

As for the privacy of the output det(M), because $det\left(M\right)=\frac{r_{1}}{t_{1}}+\frac{r_{2}}{t_{2}}$, the attacker must obtain $t_{1},t_{2}$ before computing det(M). As $t_{1}=det\left(P_{1}\right)det\left(Q_{1}\right),t_{2}=det\left(P_{2}\right)det\left(Q_{2}\right)$, computing $t_{1},t_{2}$ is equivalent to guessing $P_{1}$, $Q_{1}$, $P_{2}$, and $Q_{2}$. Thus, the probability that the attacker *A* obtains the secret output det(M) is the same as obtaining the input privacy.

Thus, we can conclude that the proposed secure outsourcing algorithm for matrix determinant is privacy-protected.

(14) $$Prob_{A}^{M}\left(Y_{1},Y_{2}\right)=\frac{1}{{\left(n!\right)}^{4}{\left(2^{\lambda }\right)}^{4n}}\to negli$$

□

#### 5.3.2. Security against Active Attacks

An active attacker (malicious) injects false computation results into the algorithm to tamper with the whole procedure.

**Theorem** **5.**  *The proposed secure outsourcing algorithm for matrix determinant is cheating-detected.*

**Proof of Theorem 5.**  There are three types of attacks with different complexity levels.

In the first attack, the attacker returns random $r_{1}^{\prime },r_{2}^{\prime },r_{3}^{\prime },r_{4}^{\prime },L_{1}^{\prime },L_{2}^{\prime },U_{1}^{\prime },U_{2}^{\prime }$ to the client with O(1) computational complexity. Obviously, $r_{1}^{\prime }\neq \prod_{i=1}^{n}L_{1}^{\prime }\left(i,i\right)U_{1}^{\prime }\left(i,i\right)$, $r_{3}^{\prime }\neq \prod_{i=1}^{n}L_{3}^{\prime }\left(i,i\right)U_{3}^{\prime }\left(i,i\right)$, and $\frac{r_{1}^{\prime }}{t_{1}}+\frac{r_{2}^{\prime }}{t_{2}}\neq \frac{r_{3}^{\prime }}{t_{3}}+\frac{r_{4}^{\prime }}{t_{4}}$. Thus, it cannot pass the verifications of Equations (5)–(7). The malicious cloud can also perform a small number of computations with O(n) computational complexity so that $r_{1}^{\prime }=\prod_{i=1}^{n}L_{1}^{\prime }\left(i,i\right)U_{1}^{\prime }\left(i,i\right)$ and $r_{3}^{\prime }=\prod_{i=1}^{n}L_{3}^{\prime }\left(i,i\right)U_{3}^{\prime }\left(i,i\right)$, which can nullify the verifications of Equations (5) and (6). However, due to the lack of $t_{1},t_{2},t_{3},t_{4}$, it still fails to pass the verification of Equation (7).

The complexity of the second type of attack is $O(n^{2.373})$. There are two ways of attacking. In the first way, the attacker computes the correct results $\sigma_{y}$, but chooses a random *a*th element on the diagonal of $L_{1}$ or $U_{1}$ and tampers with it (e.g., $L_{1}^{\prime }\left(a,a\right)=\gamma L_{1}\left(a,a\right)$). In the same way, the attacker tampers with a random *b*th element on the diagonal of $L_{3}$ or $U_{3}$ (e.g., $L_{3}^{\prime }\left(b,b\right)=\gamma L_{3}\left(b,b\right)$). Besides, the attacker also changes the $r_{1},r_{2},r_{3},r_{4}$ by $r_{1}^{\prime }=\gamma r_{1},\ldots ,r_{4}^{\prime }=\gamma r_{4}$. The above attack returns $r_{1}^{\prime },r_{2}^{\prime },r_{3}^{\prime },r_{4}^{\prime },L_{1}^{\prime },U_{1},L_{3}^{\prime },U_{3}$ to the client, which can successfully nullify the verifications of Equations (5)–(7). However, it cannot pass the verification in lines 6–17 of the VERIFY function. The verifications in lines 6–17 verify all the diagonal elements in $L_{1}$, $L_{3}$, $U_{1}$, and $U_{3}$ at least once. The nature of the verifications in lines 6–17 is to select 2n elements in the matrices $Y_{1}$ and $Y_{3}$, respectively, to verify the correctness of the diagonal elements in $L_{1}$, $L_{3}$, $U_{1}$, and $U_{3}$. As shown in Equations (15) and (16), the error in the *a*th/*b*th term in $l_{1}^{\prime }\left(a,-\right)$/$l_{3}^{\prime }\left(b,-\right)$ will be propagated to $Y_{1}^{\prime }\left(a,j\right)$/$Y_{3}^{\prime }\left(b,k\right)$, which is not equal to $Y_{1}\left(a,j\right)$/$Y_{3}\left(b,k\right)$. Thus, the forged $L_{1}^{\prime }\left(a,a\right)$ and $L_{3}^{\prime }\left(a,a\right)$ can be certainly detected.

(15) $$Y_{1}\left(a,j\right)=l_{1}\left(a,-\right)\cdot u_{1}\left(-,j\right)\neq l_{1}^{\prime }\left(a,-\right)\cdot u_{1}\left(-,j\right),\left(a\leq j\leq n\right)$$

(16) $$Y_{3}\left(b,k\right)=l_{3}\left(b,-\right)\cdot u_{3}\left(-,k\right)\neq l_{3}^{\prime }\left(b,-\right)\cdot u_{1}\left(-,k\right),\left(b\leq k\leq n\right)\;$$

In the second way, before performing the COMPUTE function, the attacker tampers with a random element in $Y_{1}$ and $Y_{3}$, respectively (Tampering with more items will be easier to detect.). Then, the attacker performs the COMPUTE function with the forged input $Y_{1}^{\prime },Y_{3}^{\prime }$, and returns the cheating result $\sigma_{y}^{\prime }=r_{1}^{\prime },r_{2},r_{3}^{\prime },r_{4},L_{1}^{\prime },U_{1}^{\prime },L_{3}^{\prime },U_{3}^{\prime }$ to the client. As the verification in lines 6–17 can only detect this attack with a probability of $\frac{2}{n}$. This way of attacking can easily nullify the verification in lines 6–17. However, as shown in Equation (17), it cannot pass the verification of Equation (7). Forging more elements in $Y_{1},Y_{3}$, or elements in $Y_{2},Y_{4}$ can also be detected by Equation (7) (e.g., the cloud returns $\sigma_{y}^{\prime }=r_{1},r_{2}^{\prime },r_{3}^{\prime },r_{4}^{\prime },L_{1},U_{1},L_{3}^{\prime },U_{3}^{\prime }$ to the client where $r_{i}^{\prime }$ is the cheating result and $r_{i}$ is the correct result).

(17) $$\frac{r_{1}^{\prime }}{t_{1}}+\frac{r_{2}}{t_{2}}\neq \frac{r_{3}^{\prime }}{t_{3}}+\frac{r_{4}}{t_{4}}$$

The complexity of the third attack is much greater than others. In order to pass all the verification, before attacking, the attacker has to make ${\left(n!\right)}^{4}$ brute-force guesses to get the 4n positions that the client verifies in $Y_{1}$ and $Y_{3}$ (lines 6–17). Then, the cloud constructs two forged matrices $Y_{1}^{\prime }$ and $Y_{3}^{\prime }$. The forged matrices $Y_{1}^{\prime }$ and $Y_{3}^{\prime }$ satisfy the condition that the elements at the detected positions remain unchanged, and the elements at other positions are changed. Meanwhile, the attacker ensures $det\left(Y_{1}^{\prime }\right)=\gamma det\left(Y_{1}\right)$, $det\left(Y_{3}^{\prime }\right)=\gamma det\left(Y_{3}\right)$. Afterwards, the cloud tampers with $r_{1},r_{2},r_{3},r_{4}$ by $r_{1}^{\prime }=\gamma r_{1},\ldots ,r_{4}^{\prime }=\gamma r_{4}$. The third attack is the only way to pass all the verifications in VERIFY with a probability of $\frac{1}{{\left(n!\right)}^{4}}$ and return a forged result $y^{\prime }=\gamma y$. The probability that the client *C* successfully detects the forged result is shown in Equation (18), which infinitely approaches 1 when *n* is large enough. Thus, we can conclude that the proposed secure outsourcing algorithm for matrix determinant is cheating-detected, when the size of the matrix is large enough.

(18) $$Prob_{C}^{forge}\left(r_{1},r_{2},r_{3},r_{4},L_{1},L_{3},U_{1},U_{3}\right)=\left(1-\frac{1}{{\left(n!\right)}^{4}}\right)\to 1$$

□

As shown in Table 6, the theoretical security of the proposed algorithm is significantly higher than the other three algorithms while using the same key space K$={\left\{1,0\right\}}^{\lambda }$. According to [4,5,6], the parameter *l* is recommended to be greater than 20. Thus, when n≥5, the proposed algorithm can achieve a high cheating detectability comparable to these three algorithms with the lowest computational cost, as demonstrated in Table 3 and Table 4. Actually, it is common to compute the determinant of the large matrices with dimensions of far-greater than 5 in DIP and machine learning.

## 6. Performance Evaluation

According to the above theoretical analysis, our algorithm can significantly reduce the local computational burden of the client. In this section, we implement the algorithm to assess its practical efficiency. The client and the cloud server functions in our experiments were conducted on the same machine, which has an Intel(R) Core(TM) i7-8550U CPU 1.80 GHz with eight cores. We implemented the proposed algorithm by Matlab and used the LAPACK [40] package to perform the LU decomposition. The communication costs between the client and the server were ignored, since the computations dominate the running time. Table 7 shows the notations used in this section.

Our goal is to reduce the client’s computational burden through outsourcing. Therefore, the ratio of time consumption without outsourcing to time consumption with outsourcing is an important measure, which is referred to as the *Acceleration Ratio* of clients.

**Figure 3:**
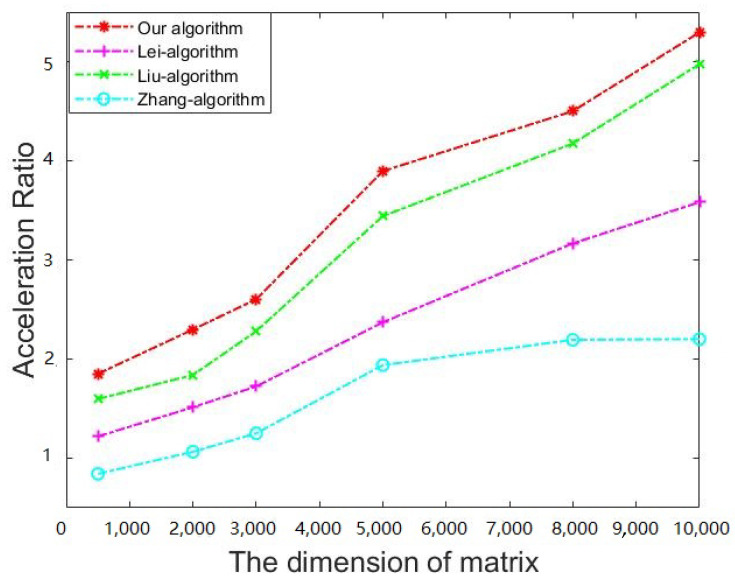
Acceleration ratio of the client.

**Figure 4:**
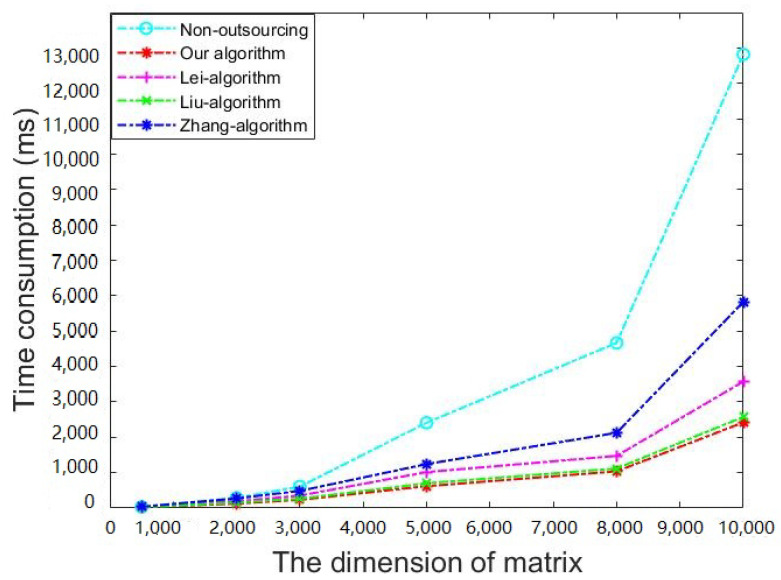
The time consumption of the client.

In our comparisons, we only considered the existing algorithms proposed for the malicious model, without considering those merely for the semihonest model, since the former model is more secure. As far as we know, the currently existing algorithms for the malicious model include only Lei’s algorithm [5], Liu’s algorithm [6], and Zhang’s algorithm [4]. In the experiment, we set the parameter *l* to 20 in the three previous algorithms, and set the parameter *m* to 500 in Lei’s algorithm. We compared the running time of every part of the algorithms on matrices of different dimensions. Table 8 and Figure 3 show that our algorithm has the highest acceleration rate on matrices of all dimensions. The time consumption on cloud in our algorithm is slightly higher than the three previous algorithms, which is not a big issue since this follows the aim of outsourcing by moving computational burden from local to cloud. As shown in Figure 4, compared with the previous algorithms and nonoutsourcing algorithms, our proposed algorithm has the lowest local computational burden. As for the security level, even if *l* is set to 20, the security of the three previous algorithms are still much lower than the proposed algorithm.

**Figure 5:**
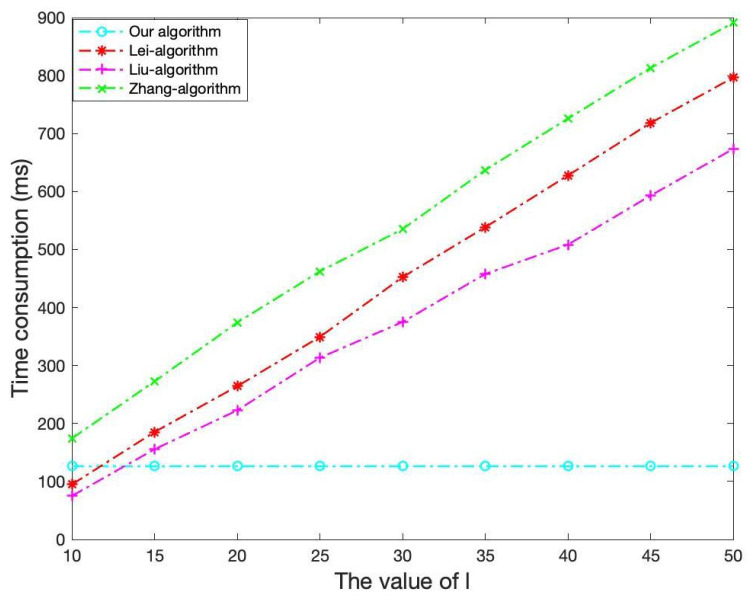
The time consumption of the client when using different parameter *l*.

In order to prove that the proposed algorithm achieves a higher security level with less local computation costs. we also compare the local running time of the proposed algorithm with the previous three algorithm on different values of parameter *l*. In this experiment, the dimension of matrix is fixed at 2000. As shown in Figure 5, because the proposed algorithm uses a new verification method, which does not involve the parameter *l*, the local running time of the proposed algorithm remains unchanged. As the parameter *l* increases, the cheating detectability of the previous three algorithms increases, since the forged results can escape local verifications with the probability of $\frac{1}{2^{l}}$, while in our algorithm the cheating detectability remains at the probability of $1-\frac{1}{{\left(2000!\right)}^{4}}$. However, the local computational burden of the three algorithms also increases significantly.

**Figure 6:**
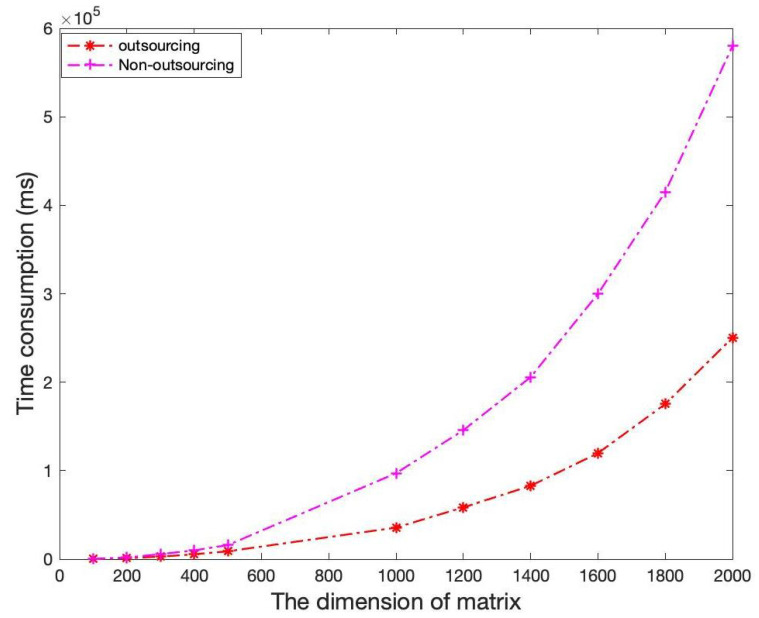
The time consumption of solving linear equations.

We also apply the proposed secure outsourcing algorithm as a basic module to the Cramer’s rule to solve linear equations. We compare the time consumption of outsourcing determinant computation and nonoutsourcing in solving linear equations of different dimensions. As shown in Figure 6, outsourcing the computations of determinant can significantly reduce the time consumption of solving linear equations, which also proves the efficiency superiority of the proposed secure outsourcing algorithm for matrix determinant computation.

## 7. Conclusions

In this paper, we propose a new secure outsourcing algorithm for matrix determinant computation under the malicious model. We also conduct theoretical analysis to prove the correctness, efficiency, privacy protection level, and cheating detectability for the proposed algorithm. In comparison with the previous algorithms in [4,5,6], the proposed algorithm achieves higher cheating detectability with less computations on the client. The previous algorithms [4,5,6] use Freivald’s method [38] for verification, which achieves a high cheating detectability by continuously increasing local computational burden. The cheating detectability of the proposed algorithm does not depend on the frequency of verification but is only related to the size of the matrix. Even in the case that the dimension of the matrix is not large enough (greater than or equal to 5), the cheating detectability of our algorithm is significantly better than the previous algorithms. For the privacy, the state-of-the-art algorithm [4] has the highest privacy, but its local computation cost usually nullifies the efficiency benefit of outsourcing when the dimension of matrix is less than 2000. Our algorithm with the lowest local computation cost achieves privacy comparable with the state-of-the-art works. We also conduct experiments to demonstrate the local efficiency superiority of the proposed algorithm. However, the theoretical analysis and experimental results also show that the proposed algorithm has a higher cloud computation cost than the three previous algorithms, which is not a big issue since this follows the aim of outsourcing by moving computational burden from local to cloud. As future work, we will study how to reduce the cloud’s computational burden in the secure outsourcing algorithms for computations of the matrix.

## Figures and Tables

**Table 1:** Efficiency and Security comparative assessment.

Algorithm	Local Multiplications	Probability of Privacy Leakage	Probability of Cheating Success
Our algorithm	$12n^{2}+12n$	$\frac{1}{{\left(n!\right)}^{4}{\left(2^{\lambda }\right)}^{4n}}$	$\frac{1}{{\left(n!\right)}^{4}}$
Lei’s algorithm [5]	$\left(2+l\right)n^{2}+2m^{2}+4mn+2n+m$	$\frac{1}{{\left(\left(n+m\right)!\right)}^{2}{\left(2^{\lambda }\right)}^{2(n+m)}}$	$\frac{1}{{\left(2\right)}^{l}}$
Liu’s algorithm [6]	$\left(2+l\right)n^{2}+3n$	$\frac{1}{{\left(n!\right)}^{2}{\left(2^{\lambda }\right)}^{2n}}$	$\frac{1}{{\left(2\right)}^{l}}$
Zhang’s algorithm [4]	$\left(10+l\right)n^{2}+6n$	$\frac{1}{{\left(n!\right)}^{2}{\left(2^{\lambda }\right)}^{10n-8}}$	$\frac{1}{{\left(2\right)}^{l}}$

**Table 2:** Symbols and implications.

Symbol	Implication
r	A vector
M	A full rank n×n matrix
det(M)	The determinant of matrix M
α←K	Choose an element α from set K randomly
λ	Security parameter
$Prob_{A}^{x}\left(\chi \right)$	The probability that attacker *A* obtains the secret input *x* using data χ
$Prob_{C}^{forge}\left(\chi \right)$	The probability that the client *C* detects the forged results forge using data χ
$M\left(i,j\right),M_{i,j}$	The element in the *i*th row and *j*th column of matrix M
$m_{i,-}$ / m(i,−)	The *i*th row of matrix M
$m_{-,j}$ / m(−,j)	The *j*th column of matrix M
$M^{T}$	The transpose of M
a·b	The inner product of vector a and vector b
$f_{LU}\left(M\right)$	The Low triangle and Up triangle decomposition of matrix M

**Table 3:** Local multiplication burden.

Function	KEYGEN	ENCRYPT	VERIFY	DECRYPT	Total
Our algorithm	8n	$8n^{2}$	$4n^{2}+4n$	1	$12n^{2}+12n$
Lei’s algorithm [5]	n+m	$2{\left(n+m\right)}^{2}$	$ln^{2}$	*n*	$\left(2+l\right)n^{2}+2m^{2}+4mn+2n+m$
Liu’s algorithm [6]	2n	$2n^{2}$	$ln^{2}$	*n*	$\left(2+l\right)n^{2}+3n$
Zhang’s algorithm [4]	2n	$10n^{2}$	$ln^{2}$	4n	$\left(10+l\right)n^{2}+6n$

**Table 4:** Local addition burden.

Function	KEYGEN	ENCRYPT	VERIFY	DECRYPT	Total
Our algorithm	8n+4	2n	$4n^{2}+2$	1	$4n^{2}+10n+7$
Lei’s algorithm [5]	2(n+m)+1	0	$2l{\left(n+m\right)}^{2}-l\left(n+m\right)$	0	$2l{\left(n+m\right)}^{2}-\left(l-2\right)\left(m+n\right)+1$
Liu’s algorithm [6]	2n+1	0	$2ln^{2}-ln$	0	$2ln^{2}-\left(l-2\right)n+1$
Zhang’s algorithm [4]	2n+1	$4n^{2}$	$2ln^{2}-ln$	0	$\left(2l+4\right)n^{2}-\left(l-2\right)n+1$

**Table 5:** Theoretical computational complexity.

Function	KEYGEN	ENCRYPT	VERIFY	DECRYPT	COMPUTE
Our algorithm	O(n)	$O(n^{2})$	$O(n^{2})$	O(1)	$O(n^{2.373})$
Lei’s algorithm [5]	O(n+m)	$O({\left(n+m\right)}^{2})$	$O(ln^{2})$	O(n)	$O(n^{2.373})$
Liu’s algorithm [6]	O(n)	$O(n^{2})$	$O(ln^{2})$	O(n)	$O(n^{2.373})$
Zhang’s algorithm [4]	O(n)	$O(n^{2})$	$O(ln^{2})$	O(n)	$O(n^{2.373})$

**Table 6:** Probability of successful attack.

Attack	Our Algorithm	Liu’s Algorithm [6]	Lei’s Algorithm [5]	Zhang’s Algorithm [4]
Privacy Leakage	$\frac{1}{{\left(n!\right)}^{4}{\left(2^{\lambda }\right)}^{4n}}$	$\frac{1}{{\left(n!\right)}^{2}{\left(2^{\lambda }\right)}^{2n}}$	$\frac{1}{{\left(\left(n+m\right)!\right)}^{2}{\left(2^{\lambda }\right)}^{2(n+m)}}$	$\frac{1}{{\left(n!\right)}^{2}{\left(2^{\lambda }\right)}^{10n-8}}$
Result cheating	$\frac{1}{{\left(n!\right)}^{4}}$	$\frac{1}{{\left(2\right)}^{l}}$	$\frac{1}{{\left(2\right)}^{l}}$	$\frac{1}{{\left(2\right)}^{l}}$

**Table 7:** Notations in experiments.

Notations	Implication
$t_{o}$	The time consumption of nonoutsourcing scheme.
$t_{s}$	The time consumption of cloud.
$t_{c1}$	The time consumption of client in key generation and encryption.
$t_{c2}$	The time consumption of client in decryption and verification.
$t_{c}$	The time consumption of client.
$\frac{t_{o}}{t_{c}}$	Acceleration ratio of client.

**Table 8:** Experimental results (time in milliseconds).

Algorithm	Dimension	$t_{o}$	$t_{c1}$	$t_{c2}$	$t_{c}$	$t_{s}$	$\frac{t_{o}}{t_{c}}$
Our algorithm	500	32.829	14.188	3.547	17.735	49.264	1.851
	2000	290.013	94.517	31.670	126.187	352.725	2.298
	3000	606.768	191.122	41.954	233.078	903.288	2.603
	5000	2410.926	470.294	148.515	618.809	2982.729	3.896
	8000	4666.675	808.776	228.117	1036.893	6163.738	4.501
	10,000	12,818.648	1960.510	459.867	2420.337	15,895.125	5.296
Lei’s algorithm [5]	500	32.829	1.747	25.184	26.931	45.775	1.219
	2000	290.013	5.528	186.026	191.554	348.904	1.514
	3000	606.768	10.951	341.002	351.953	894.031	1.724
	5000	2410.926	42.309	974.528	1016.837	2767.491	2.371
	8000	4666.675	274.662	1198.869	1473.532	5495.207	3.167
	10,000	12,818.648	796.518	2779.116	3575.634	14,175.942	3.585
Liu’s algorithm [6]	500	32.829	0.793	19.764	20.557	38.379	1.597
	2000	290.013	4.923	152.771	157.694	311.684	1.839
	3000	606.768	7.595	258.414	266.009	894.031	2.281
	5000	2410.926	30.519	669.924	701.443	2531.673	3.442
	8000	4666.675	132.731	985.303	1118.034	4951.749	4.174
	10,000	12,818.648	551.634	2024.461	2576.095	13,667.593	4.976
Zhang’s algorithm [4]	500	32.829	18.475	20.498	38.973	37.517	0.842
	2000	290.013	104.772	167.538	272.310	324.941	1.065
	3000	606.768	207.462	277.737	485.199	875.963	1.251
	5000	2410.926	522.743	720.335	1243.078	2539.492	1.939
	8000	4666.675	983.769	1142.779	2126.548	5027.820	2.194
	10,000	12,818.648	2647.539	3176.957	5824.496	12,741.742	2.201

## Data Availability

Not applicable.
